# The Impact of COVID-19 on Pediatric Healthcare Utilization and Disease Dynamics: An Observational Study From Western Odisha

**DOI:** 10.7759/cureus.27006

**Published:** 2022-07-19

**Authors:** Bijay K Meher, Isha Panda, Nihar R Mishra, Leena Das, Bhojraj Sahu

**Affiliations:** 1 Pediatrics, Sardar Vallabhbhai Patel Post Graduate Institute of Paediatrics, Srirama Chandra Bhanja Medical College and Hospital, Cuttack, IND; 2 Pediatrics, All India Institute of Medical Sciences, Kalyani, Kolkata, IND; 3 Pediatrics, Bhima Bhoi Medical College and Hospital, Balangir, IND

**Keywords:** immunisation, delivery of health care, morbidity, child health services, covid-19

## Abstract

Introduction

Children were affected by the severe acute respiratory syndrome coronavirus 2 (SARS-CoV-2) virus during the first and second waves of the coronavirus disease 2019 (COVID-19 pandemic. Although the severity was less in children, the fear of contracting SARS-CoV-2 at the hospital might have led to a delayed health-seeking attitude. The objective of the study was tailored around emergency health care utilization affecting outcomes. The study was done to compare the trend concerning the utilization of pediatric healthcare and immunization services, changes in the profile of diseases, and the outcomes between the pre-COVID-19 period and the COVID-19 period in a tertiary care hospital.

Methods

This retrospective observational study was conducted in a tertiary care hospital in western Odisha. Data were collected retrospectively from different hospital registers (outpatient register, inpatient register, and immunization records) and analyzed between the pre-COVID-19 period (April 2019 to March 2020) and the COVID-19 period (April 2020 to March 2021) with appropriate statistical procedures.

Results

There was a 60%, 49.8%, 51.1%, and 25.5% reduction in outpatient attendance, indoor pediatric admissions, nutritional rehabilitation centre admissions, and newborn admissions, respectively in COVID-19 period as compared to the pre-COVID-19 period. The pediatric bed occupancy rate was reduced by 54.5%. Unfavourable outcomes (death, left against medical advice, and referral) were significantly high in hospitalized children (24% vs. 18.1%, p < 0.001). The reduction in hospitalization due to common conditions like acute respiratory tract infections, bronchiolitis and asthma, and acute gastroenteritis during COVID-19 was 76.5%, 86.2%, and 39.5%, respectively. A higher percentage of low birth weight and preterm (<34 weeks) babies were admitted to special neonatal care unit (SNCU) during the pandemic (61.8% vs. 58%, p < 0.05; 18.9% vs. 15.8%; p < 0.05 respectively).

Conclusion

The COVID-19 pandemic and the associated lockdown led to a significant decrease in pediatric and neonatal healthcare utilization. The impact of lower care-seeking and attendance resulting in poor patient-related outcomes (malnutrition, upsurge of vaccine-preventable diseases, disease-specific hospitalization, and mortality) post-pandemic is a real threat.

## Introduction

The coronavirus disease 2019 (COVID-19), a novel viral disease caused by the severe acute respiratory syndrome coronavirus 2 (SARS-CoV-2) was first reported in Wuhan, China, in December 2019 [1]. In India, the first case of COVID-19 was detected on January 27, 2020. As of May 30, 2021, two distinct waves of the pandemic with 27.9 million confirmed infections and 320,000 deaths have been reported from India [2]. Social distancing and the use of masks and sanitizers were introduced to prevent the spread of infection. To reduce the rate of infection and to reduce the burden on healthcare systems, a nationwide lockdown was initiated in India on March 25, 2020. Dedicated COVID care centres and COVID hospitals were created with the idea to segregate COVID-19 patients.

India witnessed the first wave of the COVID-19 pandemic in June 2020, which lasted till December 2020, with a peak in September 2020. The arrival of the second wave was signalled by an increasing number of cases from the first week of March 2021. Though children were affected by the virus with less severity, transportation difficulty due to lockdown and the fear of contracting the infection at the health care facility modified the health care use of pediatric patients. A study by Joy, et al. [3] revealed a decrease of 65% in face-to-face consultations that coincided with the lockdown in the United Kingdom. Similar decreases in the utilization of various healthcare services during the COVID-19 pandemic or others, such as SARS, have been confirmed by several other studies [4-8]. Physician consultations, specialist referrals, and hospital admissions decreased tremendously during the COVID-19 lockdown [9-12].

Evidence on the impact of COVID-19 and the lockdown measures on the pediatric healthcare utilization and disease rate in India is limited. Therefore, this study aimed to assess the trend concerning the utilization of pediatric and neonatal healthcare services, including immunization, and change in incident diseases and outcomes during the COVID-19 pandemic compared to the pre-pandemic period in a tertiary care hospital.

## Materials and methods

Study design and subjects

This observational analytical cross-sectional study was conducted in a tertiary care hospital in western Odisha, India. The hospital, which serves as a referral centre for five districts, has a maternal and child health (MCH) unit with 30 beds in the pediatric ward, 24 in the special neonatal care unit (SNCU), and 10 in the nutritional rehabilitation centre (NRC). One urban immunization clinic is attached to the MCH unit that immunizes all inborn and a few outborn babies. This hospital has a separate ward for suspected and confirmed COVID-19 patients and data from the COVID hospital were not analyzed. Routinely collected health services data from different health care units (outpatient record, indoor admission record, special neonatal care unit database, nutritional rehabilitation centre record, immunization clinic records) from April 2019 to March 2021 were analyzed. Two time periods were considered: the pre-COVID-19 period (1 April 2019 to 31 March 2020) and the COVID-19 period (1 April 2020 to 31 March 2021). Patients were neither involved in defining the research questions nor the outcome measures. The design of the study does not involve any participation of patients or any dissemination of results.

Data collection and variables

Data on the health care utilization and outcome were collected from the hospital registers (outpatient register, inpatient register, NRC register, SNCU register, and immunization register). The variables collected every month were: outpatient attendance (the emergency attendance was also included under this); pediatric indoor admission, indication, and outcome; NRC admission and outcome; delivery and mode of delivery, stillbirth; neonatal admission in SNCU, indication, and outcome, and immunization. Records collected from the registers were cross-checked with the SNCU database. The demographic details, disease pattern, and the outcome in the neonatal population like gestational age, duration of stay, birth weight, age at death, disease conditions, the ratio between live birth and stillbirth, inborn and outborn, and male and female were compared between the pre-COVID-19 and COVID-19 periods. Neonates born before completing 37 weeks were considered preterm, and those beyond completing 42 weeks were considered post-term. The utilization of immunization services at different age groups was compared. the percentage change in outpatient attendance, pediatric admission, NRC admission, SNCU admission, immunization visits, and pediatric disease proportions among inpatient department (IPD) admission between COVID-19 and pre-COVID-19 periods were calculated. The linear trend of outpatient department (OPD)** **attendance, IPD admission, death, and bed occupancy was plotted as a scatter diagram.

Operational definitions

Bed occupancy rate (BOR%) was calculated as the number of beds effectively occupied by a patient for curative care divided by the number of beds available for curative care in the hospital multiplied by a defined period (365 days), with the ratio multiplied by 100.

BOR% = {(number of inpatient days for a given period)/(available beds × number of days in the period)] × 100

Acute respiratory tract infection (ARTI) was defined as any child presenting with fever, running nose, cough, cold, coryza, throat pain, and other features of upper respiratory tract involvement.

Statistical analysis

The data collected in the process were scrutinized, codified, and recorded in a predesigned case report format (CRF). Data validation was done manually by two separate persons not involved in the study. Continuous data were expressed in mean (SD); categorical data were expressed in proportions. Data normalcy testing of continuous data was done by the Shapiro-Wilk test and no transformation was required. All the relevant descriptive and inferential statistics were done by SPSS Statistics version 25.0 (IBM Corp., Armonk, USA). Pearson's Chi-square test was applied to calculate the strength of association. Odds ratio (OR) with a 95% confidence interval (CI) was calculated for getting an association between pre-COVID-19 and COVID-19 periods about hospital visits due to various diseases by Dxt v1.0 (Biostatistics Resource and Training Centre, Christian Medical College Vellore, India). For all statistical purposes, a p-value < 0.05 was considered significant.

## Results

**Figure 1:**
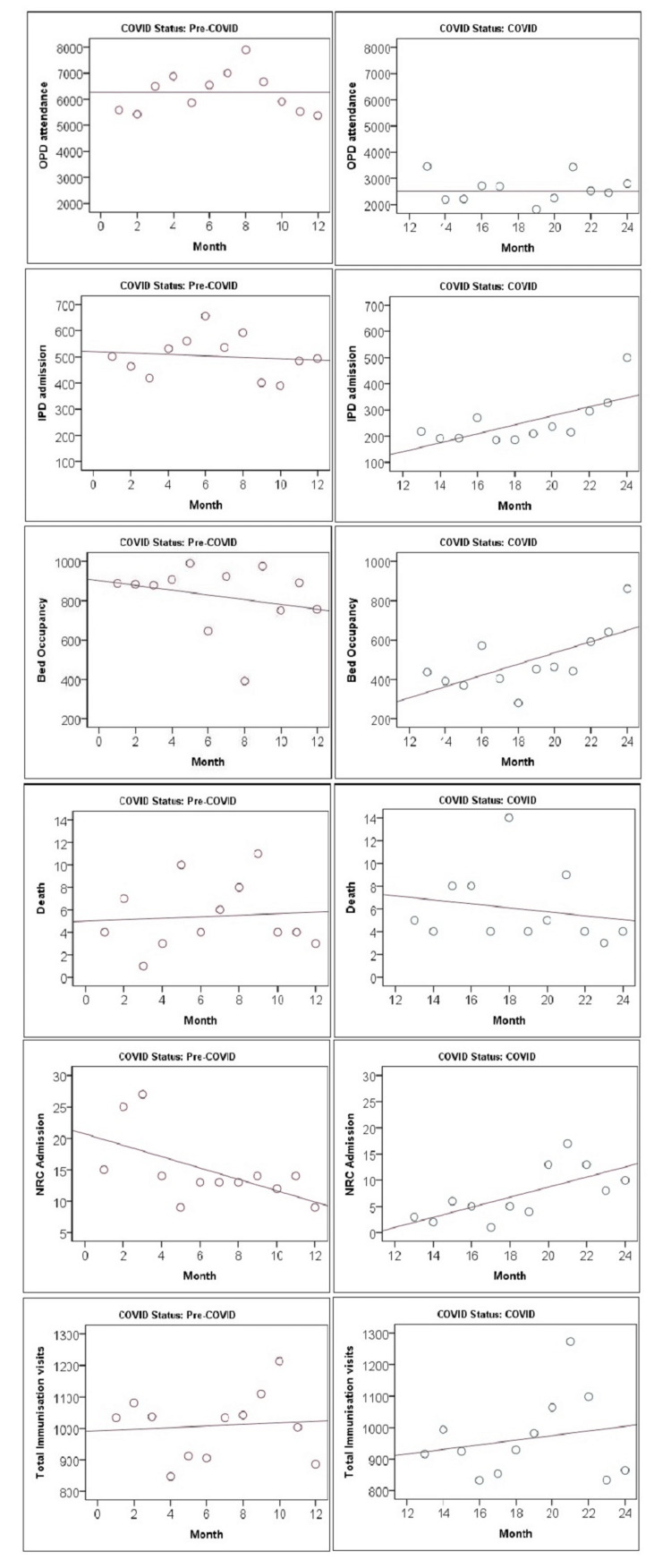
Scatter plot of OPD attendance, IPD admission, death, bed occupancy, death, NRC DHH admission and total immunization visits on month by pre-COVID-19 and COVID-19 period. IPD: inpatient department; OPD: outpatient department; NRC: nutritional rehabilitation centre; DHH: district headquarter hospital.

**Figure 2:**
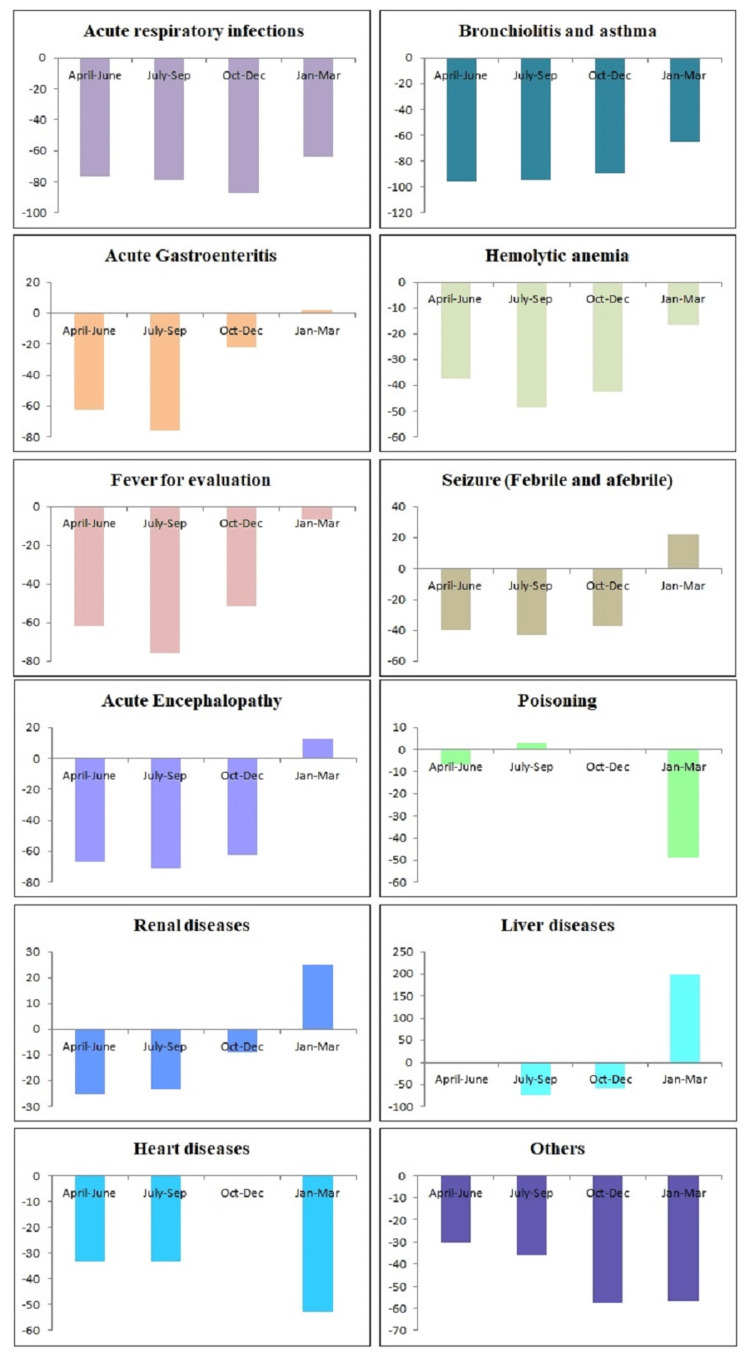
Percentage difference of pediatric diseases between COVID-19 and pre-COVID-19 periods among IPD admissions IPD: inpatient department

Table 1 shows the reduction of health care utilization during the COVID-19 period in outpatient, inpatient, NRC, SNCU, and immunization clinics in terms of percentage difference. However, a scatter plot shows the trend of OPD attendance, IPD admission, death, bed occupancy, death, NRC admission, and total immunization visits per month during the pre-COVID-19 and COVID-19 period (Figure 1). Table 2 shows the percentage reduction of different pediatric diseases needing hospitalization during the COVID-19 period as compared to the pre-COVID-19 period. The comparative trend in corresponding periods of the year between pre-COVID-19 and COVID-19 is depicted in Figure 2. July-December 2020 was the period maximally affected, with an improving trend in January-March 2021. Table 3 compares the delivery characteristics, neonatal parameters, and disease strength between the pre-COVID-19 and COVID-19 periods.

**Table 1:** Pediatric health care utilization and outcome: comparison between the pre-COVID-19 and the COVID-19 periods * Percentage change, ^€^ n (%); ^$^ left against medical advice, ^ß^ left before completion of 14 days of hospitalization, ^@^ p < 0.05 is considered as statistically significant

Health care Unit	Pre-COVID-19 (Apr 2019-Mar 2020)	COVID-19 (Apr2020-Mar 2021)	^*^ % change / p value ^@^
*Outpatient attendance (n)	75187	30094	- 60.0 ^*^
Paediatric Inpatient			
*Total Admission (n)	6027	3025	- 49.8^*^
^€^Outcome:			
Discharge	4530 (82.0)	2186 (76.0)	
Death	65 (1.2)	72 (2.5)	< 0.001
Refer	442 (8.0)	298 (10.4)	
LAMA^$^	490 (8.9)	319 (11.1)	
*Bed occupancy rate (%)	119.9	54.6	- 54.5^*^
Nutrition Rehabilitation Centre			
*Total Admission (n)	178	87	- 51.1^*^
^€^Outcome:			
Discharge	161(90.4)	80 (92.0)	
Refer	11(6.2)	7 (8.0)	0.498
Defaulter ^ß^	6 (3.4)	0 (0)	
Special Neonatal Care Unit			
*Total Admission (n)	3036	2262	25.5^*^
^€^Outcome:			
Discharge	1955 (64.1)	1370 (62)	
Referred	607 (19.9)	446 (20.2)	0.277
LAMA$	91(3)	79 (3.6)	
Death	396 (13)	315(14.3)	
*Immunisation visits			
At Birth	10870	10300	- 5.2^*^
At 6 weeks	267	282	5.6^*^
At 10 weeks	271	270	- 0.4^*^
At 14 weeks	269	274	1.9^*^
At 09 months	222	218	- 1.8^*^
At 16-18 months	205	218	6.3^*^

**Table 2:** Paediatric disease percentages among IPD Admission: comparison between corresponding periods in pre-COVID-19 (2019-2020) and COVID-19 (2020-2021) ^@^ acute respiratory tract infections; ^#^ acute rheumatic fever, juvenile idiopathic arthritis, acute leukemia, Wilson's disease, vasculitis; * percentage change; IPD: inpatient department.

Conditions	April-June 2019	April-June 2020			July-Sep 2019	July-Sep 2020		Oct-Dec 2019	Oct-Dec 2020		Jan-Mar 2020	Jan-Mar 2021		*Pooled Diff. (%)	
	Pre-Covid	Covid		*Diff. (%)	Pre-Covid	Covid	*Diff. (%)	Pre-Covid	Covid	*Diff. (%)	Pre-Covid	Covid	*Diff. (%)		
ARTI^@^	240	57		-76.3	184	39	-78.8	406	52	-87.2	177	64	-63.8	-76.5	
Bronchiolitis and asthma	87	4		-95.4	42	2	-95.2	148	16	-89.2	71	25	-64.8	-86.2	
Acute Gastroenteritis	300	113		-62.3	349	84	-75.9	160	125	-21.9	415	424	2.2	-39.5	
Hemolyticanemia	286	179		-37.4	392	202	-48.5	319	183	-42.6	272	228	-16.2	-36.2	
Fever for evaluation	192	74		-61.5	422	104	-75.4	213	103	-51.6	123	115	-6.5	-48.8	
Seizure (febrile and afebrile)	96	58		-39.6	111	63	-43.2	103	65	-36.9	81	99	22.2	-24.4	
Acute Encephalopathy	12	4		-66.7	14	4	-71.4	16	6	-62.5	8	9	12.5	-47	
Poisoning	30	28		-6.7	33	34	3	23	23	0	39	20	-48.7	-13.1	
Renal diseases	12	9		-25	13	10	-23.1	11	10	-9.1	8	10	25	-8.1	
Liver diseases	6	6		0	11	3	-72.7	7	3	-57.1	1	3	200	17.6	
Heart diseases	9	6		-33.3	3	2	-33.3	10	10	0	17	8	-52.9	-29.9	
Others^#^	56	39		-30.4	102	65	-36.3	59	25	-57.6	109	47	-56.9	-45.3	

**Table 3:** Delivery characteristics, neonatal parameters, and disease strength: pre-COVID-19 and COVID-19 periods * Percentage change; ^€^ n (%); ^#^p < 0.05 is considered as statistically significant; M: male; F: female: SNCU: special neonatal care unit

Factors	Pre-COVID-19 (Apr 2019-Mar 2020)	COVID-19 (Apr 2020-Mar 2021)	*% change/ 'p-value
*Total Deliveries	10791	9729	-9.8^*^
*Caesarean deliveries	3156	3074	-2.6^*^
Live birth: Still birth	27.3: 1	24.1: 1	0.093
Inborn: Outborn	1: 1	0.84: 1	0.002^#^
M: F	1.4: 1	1.6: 1	0.068
*Total SNCU Admissions	3036	2262	25.5
^€^Birth weight (gm)			
≥2500	1275 (42.0%)	865 (38.2%)	0.033^#^
<2500	1761 (58.0%)	1397 (61.8%)	
^€^Gestational Age (wks)			
≥34	2556 (84.2%)	1835 (81.1%)	
<34	480 (15.8%)	427 (18.9%)	
^€^Duration of stay (days)			
<1	300 (9.9%)	240 (10.6%)	0.026^#^
1-3	1222 (40.2%)	816 (36.1%)	
≥4	1514 (49.8%)	1206 (53.2%)	
^€^Total	3036 (100%)	2262 (100%)	
^€^Age at death			
<1day	49 (12.4%)	55 (17.5%)	0.099
1-6day	301(76.0%)	218 (69.2%)	
>6day	46 (11.6%)	42 (13.3%)	
Total	396 (100%)	315 (100%)	
^€^Disease conditions			
Mild birth asphyxia	873 (28.7%)	714 (31.5%)	0.000^#^
Moderate-severe birth asphyxia	384 (12.7%)	339 (15.0%)	
Sepsis	441 (14.5%)	309 (13.7%)	
Respiratory distress syndrome	90 (3.0%)	68 (3.0%)	
Meconium aspiration syndrome	63 (2.1%)	34 (1.5%)	
Jaundice requiring phototherapy	323 (10.7%)	22 (1.0%)	
Major Congenital Malformation	56 (1.8%)	44 (2.0%)	
Others	806 (26.5%)	732 (32.3%)	
Total	3036 (100%)	2262 (100%)	

Among children between COVID-19 and pre-COVID-19 periods, statistically significant difference were found in the hospital visits due to acute respiratory tract infection (ARTI) (Pearson's chi-square 162.62; OR 0.37; 95% CI 0.32, 0.44, p < 0.001), bronchiolitis and asthma (Pearson's chi-square 85.95; OR 0.26; 95% CI 0.19, 0.35, p < 0.001), acute gastroenteritis (AGE) (Pearson's chi-square 22.41; OR 1.30; 95% CI 1.16, 1.42, p < 0.001), haemolytic anemia (Pearson's chi-square 30.10; OR 1.33; 95% CI 1.20, 1.47, p < 0.001), fever for evaluation (Pearson's chi-square 11.35; OR 0.80; 95% CI 0.71, 0.91, p < 0.001), and seizure disorders (Pearson's chi-square 25.09; OR 1.50; 95% CI 1.28, 1.76, p < 0.001).

Among children between COVID-19 and pre-COVID-19 periods, insignificant difference were found in the hospital visits due to acute encephalopathy (Pearson's chi-square 0.12; OR 0.91; 95% CI 0.56, 1.50, p > 0.05), poisoning (Pearson's chi-square 15.87; OR 1.70; 95% CI 1.30, 2.20, p < 0.001), renal diseases (Pearson's chi-square 6.93; OR 1.78; 95% CI 1.15, 2.74, p < 0.01), liver diseases (Pearson's chi-square 0.30; OR 1.19; 95% CI 0.63, 2.27, p > 0.05) and heart diseases (Pearson's chi-square 1.27; OR 1.33; 95% CI 0.80, 2.19, p > 0.05).

The association of hospital visit due to other diseases (acute rheumatic fever, juvenile idiopathic arthritis, acute leukemia, Wilson's disease, and vasculitis) among children between COVID-19 and pre-COVID-19 periods was statistically not significant (Pearson's chi-square 0.64; OR 1.08; 95% CI 0.89, 1.30, p < 0.001).

## Discussion

This study provides information on the utilization of pediatric and neonatal health care services and the recognition of disease trends in a tertiary care hospital during the COVID-19 disease and the mandated lockdown (2020-2021). As compared to the pre-COVID-19 period (year preceding COVID-19 and mandated restriction measures), there was a decrease in OPD consultation, inpatient hospital admission, NRC admission, deliveries, neonatal admissions, and immunization visits. During the rapid rise of the COVID-19 pandemic, a reduction in the number of patients presenting to emergency departments has been observed [6, 13, 14]. There is around a 50% reduction in OPD attendance, indoor admission, and NRC admission. The bed occupancy rate in the pediatric indoor facility showed a 54.5% reduction during the COVID-19 period. Kruizinga, et al. reported a similar reduction of 59% in general pediatric care and a 56% reduction in ED visits and admission [14].

Closure of schools and decreased contact between the children might have contributed to a decrease in communicable diseases. We found a significant reduction in respiratory infections during COVID-19 and lockdown (76.5% and 86.2% reduction in ARI, and asthma and bronchiolitis, respectively). Kruizinga, et al. reported a 77% reduction in communicable diseases during the COVID-19 pandemic [14]. There were 63% fewer odds of hospital visits due to ARI among children during COVID-19 as compared to the pre-COVID-19 period. The odds of hospital visits due to bronchiolitis and asthma among children during COVID-19 were 74% less as compared to the pre-COVID-19 period. Several studies reported a similar effect of 76-84% reduction in ED visits due to asthma [5, 15]. This could be partly due to the less exposure to outdoor allergens along with avoidance of health seeking for fear of contracting the COVID-19 infection. There were 20% fewer odds of hospital visits due to ‘fever for evaluation’ among children during COVID-19.

However, for acute encephalopathies, and chronic liver and heart disease, hospitalizations were not reduced during COVID-19, this trend may be explained based on chronicity per se. There were approximately 1.3 times higher odds of hospital visits due to acute gastroenteritis and hemolytic anemia among children during the COVID-19 period as compared to the pre-COVID-19 period. The cause of admission was beyond the scope of this study, however, there could be many factors for this effect. The use of oral rehydration salts (ORS) and zinc early could have prevented severe dehydration and hospitalization in acute gastroenteritis. Similarly, delayed health-seeking behaviour in transfusion-dependent hemolytic anemia during COVID-19 might have led to severe anemia, its complication, and hospitalization. Also, there were 1.5 times higher odds of hospital visits due to seizure disorder, poisoning, and chronic renal cases among children during the COVID-19 period as compared to the pre-COVID-19 period. Fear of contacting the SARS-CoV-2 infection in hospital might have refrained parents to seek early hospitalization. Several countries have reported higher admission/ED visit ratios and worse triage codes at ED visits associated with a higher acuity of disease [16, 17].

Unfavourable outcomes (death, LAMA, and referral) were significantly high (24% vs. 18.1%) in pediatrics indoors during COVID-19, which reflects the higher acuity of disease at presentation. Several reports regarding collateral harm due to delayed presentation have been reported in the Netherlands during the COVID-19 lockdown [18].

However, pregnancy and delivery were not affected significantly by the health-seeking behaviour of people. The rate of stillbirth during the COVID-19 period was comparable to the pre-COVID-19 period. The reduction of deliveries during COVID-19 was marginal (9.8%), with an insignificant change in lower segment caesarean section (LSCS)** **delivery. The change in total deliveries, caesarean delivery, and stillbirth was marginal, which was in favour of utilizing emergency health care at appropriate times even during the COVID-19 pandemic and mandated lockdown. A significant increase in outborn admission in SNCU compared to inborn admission could be due to the paralysis of peripheral health centres during COVID-19. However, admission to SNCU was reduced by 25.5% with a higher percentage of low birth weight (< 2500 gm.) and preterm (< 34 weeks) admissions during the COVID-19 period. There is no significant change in the outcome of sick admitted neonates between COVID-19 and the pre-COVID-19 era (14.3% vs. 13%). Since the study excluded pregnancy with COVID-19, it does not reflect the effect of COVID-19 during pregnancy and childbirth. There was only a marginal change in immunization clinic visits during the COVID-19 period as compared to the pre-COVID-19 period.

The study shows that there was a decrease in OPD attendance immediately after the lockdown measures. The decrease in OPD attendance may be due to the fear of contracting the disease in the hospital and the restricted movement of private and public vehicles during the lockdown. The online consultation and the public message of skipping the non-emergency hospital visits and regular follow-ups could be adding to the above effect. The monthly average IPD admission was low compared to the pre-COVID-19 period but showed an increasing trend after the first wave (January 2021- March 2021). Pediatric hospitalization was maximally affected during July-December 2020, coinciding with the first wave of the pandemic.

Limitations

This study has several limitations. The present study was done and its conclusion was derived based on the hospital data of a single tertiary care centre. A multicentre stratified study will reflect the actual utilization of the resources and future implications in the resurgence of the childhood condition post-COVID-19 due to lack of utilization of healthcare resources. The profiles of patients attending outpatient were not analyzed. Data on the utilization of tele-consultation services during the study period were not collected. The data from the COVID-19 hospitals during the study period were not analyzed in the present study, however, the pediatric and neonatal admission to COVID-19 hospitals was much less compared to adult admissions during the study period. Whether the downturn detection of incident disease will be partly compensated by an upturn post-COVID-19 or will it result in adverse patient-related outcomes (higher disease-specific hospitalization and mortality) is beyond the scope of the present study.

## Conclusions

The COVID-19 pandemic and the associated lockdown had a significant impact on pediatric and neonatal healthcare utilization. There was a large reduction in communicable diseases and asthma-related admissions. Care utilization for non-infectious diagnoses was marginally reduced. Perinatal care was not compromised in the study site, which was in favour of utilizing emergency health care at appropriate times even during the lockdown and the COVID-19 pandemic. A significantly high percentage of low birth weight and preterm (<34 weeks) babies needed admission. The impact of the poor utilization in terms of poor patient-related outcomes (malnutrition, disease-specific hospitalization, and mortality) post-pandemic is a real threat.
